# *ALKBH2* inhibition alleviates malignancy in colorectal cancer by regulating *BMI1*-mediated activation of *NF-κB* pathway

**DOI:** 10.1186/s12957-020-02106-0

**Published:** 2020-12-10

**Authors:** Bingxin Ke, Kejun Ye, Shaobing Cheng

**Affiliations:** Department of Colorectal Surgery, the First Affiliated Hospital, College of Medicine, Zhejiang University, No. 79, Qingchun Road, Xiacheng District, Hangzhou City, 310003 Zhejiang Province China

**Keywords:** Colorectal carcinoma, alkB homolog 2, Alpha-ketoglutarate dependent dioxygenase, B cell-specific Moloney murine leukemia virus integration site 1, NF-κB pathway

## Abstract

**Background:**

The alkB homolog 2, alpha-ketoglutarate-dependent dioxygenase (ALKBH2) gene is involved in DNA repair and is expressed in different types of malignancies. However, the role of ALKBH2 in colorectal carcinoma (CRC) remains unclear. This study aimed to explore the potential mechanism of ALKBH2 and its function in CRC.

**Methods:**

The expression levels of ALKBH2 in CRC tissues and cells were determined by qRT-PCR. Following that, the role of ALKBH2 in cell proliferation, invasion, and epithelial-mesenchymal transition (EMT) in CRC cells (Caco-2 and LOVO) were assessed by Cell Counting Kit-8 (CCK-8), transwell assays, and Western blotting, respectively. The effect of ALKBH2 on B cell-specific Moloney murine leukemia virus integration site 1 (BMI1) and downstream NF-κB pathway was determined by Western blotting and luciferase reporter assay.

**Results:**

The expression of ALKBH2 was significantly upregulated both in CRC tissues and cells. Further experiments demonstrated that reduction of ALKBH2 suppressed Caco-2 and LOVO cell proliferation and invasion. Moreover, ALKBH2 knockdown also suppressed EMT, which increased E-cadherin expression and reduced N-cadherin expression. Besides, ALKBH2 silencing inhibited BMI1 expression and reduced nuclear accumulation of the NF-κB p65 protein, as well as the luciferase activity of NF-κB p65. Upregulation of BMI1 reversed the effect of ALKBH2 knockdown on the proliferation and invasion in CRC cells.

**Conclusions:**

Our findings suggest that suppression of ALKBH2 alleviates malignancy in CRC by regulating BMI1-mediated activation of NF-κB pathway. ALKBH2 may serve as a potential treatment target for human CRC.

## Background

Colorectal carcinoma (CRC) is one of the most common malignant tumors worldwide [1], ranking the third highest incidence of all malignant tumors and the fourth highest mortality rate after lung cancer, liver cancer, and gastric cancer [2]. Study demonstrated that metastasis is the leading cause of death in CRC patients [3]. Approximately 65% of patients with CRC are affected by metastasis at the time of diagnosis or will develop metastasis later; the liver is mostly involved (40–60%) in a synchronous or metachronous manner, but only 25% of hepatic metastatic lesions are resectable at the first evaluation [4, 5]. Clinically, micrometastasis failed to be detected in most patients undergoing radical surgery. This is one of the primary causes that lead to failed surgical therapy and subsequent tumor recurrence [6]. The occurrence of CRC is a complex biological process caused by the imbalance of various tumor-associated genes. Thus, in-depth knowledge of the molecular mechanism of CRC occurrence and development is of great scientific significance and clinical value for early diagnosis, diacritics, and target therapy of CRC.

AlkB protein is a repair dioxygenase that reverses alkyl DNA bases by an α-ketoglutarate/Fe(II)-dependent mechanism [7]. The ALKBH protein family is highly expressed in all kinds of human malignant tumors and has been implicated in tumor progression and development. To date, eight mammalian homologues, human AlkB homologues 1–8, have been identified by bioinformatic analysis [8, 9]. Only two of the corresponding human proteins, hABH2 and hABH3, have been shown to possess a similar repair activity as AlkB from *Escherichia coli* [10]. ALKBH2 is a housekeeping enzyme, which protects the mammalian genome from 1-meA damage by repairing the damage in double-stranded DNA (dsDNA) [11]. Wu et al. reported that ALKBH2 knockdown enhanced the cisplatin sensitivity in H1299 lung carcinoma cells [11]. Fujii and his colleagues demonstrated that ALKBH2 is overexpressed in bladder cancer [12]. Moreover, ALKBH2 knockdown restrains epithelial to mesenchymal transition (EMT), which increases E-cadherin expression and reduces vimentin expression [12]. The DNA repair enzyme ALKBH2 is implicated in both tumorigenesis and resistance to chemotherapy in cancers [13]. However, the effects of ALKBH2 on the progression of CRC and the molecular mechanisms underlying metastasis remain unclear.

The B cell-specific Moloney murine leukemia virus integration site 1 (BMI1) protein is one of the polycomb-group (PcG) family of proteins [14]. Recently, researchers have found that BMI-1 oncogene-driven pathway plays an important part in tumor development and metastases [15], and BMI-1 overexpression has been demonstrated in many carcinomas. Song et al. have proven that upregulation of BMI1 increases the motility and invasiveness of human nasopharyngeal epithelial cells, while BMI1 knockdown decreases cell motility [16]. In addition, in vivo studies reported the upregulation of BMI1 in cancer tissues and are associated with remote metastases of gastric [17], ovarian [18], breast [19], pancreatic [20], and primary hepatocellular carcinoma (HCC) [21]. Furthermore, BMI1 overexpression has also been found in patients with lymphoma [22], chronic myeloid leukemia [23], and acute myeloid leukemia [24].

Here, the biological roles of ALKBH2 in CRC were investigated. Our findings showed that ALKBH2 is significantly over-expressed in CRC tissues and cells. Besides, ALKBH2 knockdown inhibits CRC cell (Caco-2 and LOVO) proliferation, invasion, and EMT. Further studies have found that ALKBH2 affects the proliferation and aggressive progression of CRC cells through regulating BMI1 expression as well as the downstream NF-κB pathway. Thus, these results suggest that ALKBH2 stimulates CRC metastasis and invasion by regulating the activation of BMI1-mediated NF-κB pathway, which may be an oncogene involved in the evolution and invasive development of CRC.

## Methods

### Patients and specimens

Colorectal cancer tissues (*N* = 39) and para-carcinoma tissues (> 2 cm away from the tumor, *N* = 33) were obtained from CRC patients, who underwent surgical operations in the First Affiliated Hospital, College of Medicine, Zhejiang University. All patients were diagnosed by histology, and none of the patients received chemotherapy or radiotherapy before operation. Tissues were preserved in liquid nitrogen for subsequent experiments. This study was approved by the Research Ethics Committee of the First Affiliated Hospital, College of Medicine, Zhejiang University. Consents were obtained from all participants before the study commenced. This study was performed in accordance with the World Medical Association Declaration of Helsinki [25].

### Cell culture

Human CRC cell lines (Caco-2, SW480, HCT15, HT-29, SW837, and LOVO) and normal colorectal epithelium cell line (CCD-18Co) [26] were received from the Institute of Biochemistry and Cell Biology of the Chinese Academy of Sciences (Shanghai, China). Cells were cultured in RPMI 1640 or DMEM medium (GIBCO-BRL) containing 10% fetal bovine serum (FBS), 100 U/ml penicillin, and 100 mg/ml streptomycin and incubated at 37 °C, 5% CO_2_. After 2–3 stable generations, logarithmic-phased cells were used in subsequent experiments.

### Cell transfection

Cells were transfected with specific shRNA oligonucleotides using Lipofectamine® 2000 (Invitrogen, Carlsbad, CA, USA) according to the manufacturer’s protocols. The ALKBH2-targeting shRNA (shALKBH2) and the negative non-targeting control, shNC, used in this study were synthesized by Invitrogen. The sequence of shALKBH2 is as follows (5′ to 3′): CCGGGTGCTCATCAACAGGTATAAACTCGAGTTTATACCTGTTGATGAGCACTTTTTG. To construct BMI1-overexpressing plasmids, BMI1 cDNA was cloned into p-MSCV-IRES-GFP. For transfection, cells were seeded in 6-well plates and cultured for 24 h until cells reached 70–80% confluence. Then, cells were transfected with 2 μg of plasmid using Lipofectamine® 2000 (Invitrogen) following the manufacturer’s instructions.

### RNA extraction and qRT-PCR

Total RNA was extracted from tissues or cultured cells using the TRIzol reagent (Invitrogen, USA), according to manufacturer’s protocols. A First Strand cDNA Synthesis Kit (Takara, Dalian, China) was used for reverse transcription with random primers. qRT-PCR was carried out using SYBR Premix Ex Taq (Takara, USA), following the manufacturer’s instructions on an ABI 7500 real-time PCR system (Appiled Biosystems, USA). The primer sequences are shown in Table 1. GAPDH expression was used as a reference. Three experimental triplicates were performed.

**Table 1:** The specific primer pairs used in quantitative real-time RT-PCR

Genes	Primers	Sequences
ALKBH2	Forward	5′-GACTGGACAGACCTTCAAC-3′;
	Reverse	5′-AGGAGACAGAGGCAATGG-3′
BMI1	Forward	5′-ATGCATCGAACAACGAGAATCAAGATCACT-3′
	Reverse	5′-TCAACCAGAAGAAGTTGCTGATGACCC-3′
GAPDH	Forward	5′-GGAGCGAGATCCCTCCAAAAT-3′
	Reverse	5′-GGCTGTTGTCATACTTCTCATGG-3′

### Cell proliferation assay

Cell proliferation was assessed by Cell Counting Kit-8 assays (CCK-8; Dojindo Molecular Technologies, Japan). The transfected cells (Caco-2 and LOVO) were cultured in 96-well plates (5 × 10^3^cells/well). Then, each well was incubated with CCK-8 reagent at 0, 24, 48, 72, and 96 h, and cells were further cultured for 2 h at 37 °C. Absorbance at 450 nm was read using a microplate reader. All tests were repeated three times.

### Cell migration and invasion assays

Cell migration analysis was performed using the transwells (8-mm pore size, Corning, USA). For invasion assays, the upper surface of the membrane was coated with Matrigel (BD Biosciences, USA) at 37 °C for 4 h, whereas for the migration assay, the top chamber uncoated. A total of 2 × 10^4^ cells in 200 μL of serum-free medium were seeded onto the upper chamber of transwell plates. The lower chamber was supplemented with medium containing 10% FBS. After 24 h of incubation, cells in the upper chambers were removed by a cotton swab, whereas cells that migrated into the lower chamber were immobilized by cold methanol and stained with 0.1% crystal violet. All migrated and invaded cells were visualized under a light microscope and manually counted in at least ten random regions for each transwell membrane.

### Western blotting

Proteins were extracted from cells using RIPA buffer (Beyotime, Haimen, China) containing 1% protease inhibitor cocktail (Roche, Diagnostics GmbH, Mannheim, Germany) and phenylmethylsulfonyl fluoride (PMSF; Roche). Then, proteins were separated by SDS-PAGE and transferred onto nitrocellulose membranes (Sigma-Aldrich). Membranes were blocked with 5% milk in Tris-buffered saline (TBS) for 1 h at 25 °C, followed by incubation with primary antibodies: anti-ALKBH2 (1:1000; Invitrogen), anti-TGFβ2, anti-Smad3, anti-pSmad3, anti-N-cadherin, anti-E-cadherin, anti-Vinmentin, anti-Snail1, and anti-Slug (all 1:1000; Cell Signaling Technology) at 4 °C overnight. Next, membranes were incubated with secondary antibodies (HRP-conjugated 1:5000; Santa Cruz Biotechnology) at 37 °C for 2 h. Lastly, protein bands of interest were visualized and imaged by ECL method using the Bio-Rad imaging system. Protein band intensities were quantified using ImageJ software [27]. For nuclear protein extraction, nuclear and cytoplasmic proteins were separated using Solarbio.

### Immunohistochemistry

Immunohistochemistry (IHC) was performed for normal tissues as previously described [28, 29]. Briefly, paraffin sections were incubated with anti-ALKBH2 (1:100; Santa Cruz Bio, Bath, UK) at 4 °C overnight, followed by 30-min incubation at 25 °C with goat anti-rabbit Envision System Plus-HRP (Dako Cytomation). After washing with PBS for 3 times, samples were incubated with DAB for 1 min and counterstained with Mayer hematoxylin, dehydrated, and fixed. PBS was used as the negative control.

### Luciferase reporter assay

Luciferase reporter assay was used to assess the expression of target genes. First, pRL-TK renilla luciferase plasmids, firefly luciferase reporter plasmids, and pNF-kB-luciferase plasmid were co-transfected in cells. Luciferase activity was detected using a Dual-Luciferase Reporter Assay System (Promega, Madison, WI, USA). The transfection efficiency data were normalized by differentiating firefly luciferase activity with renilla luciferase activity.

### Statistical analysis

SPSS 17.0 software package (IBM, Chicago, IL) was used for statistical analysis. Results are shown as mean ± standard deviation (SD). When comparing two groups, Student’s *t* test was used to compare the differences, whereas one-way ANOVA analysis was used when comparing multiple groups. *P* value < 0.05 was considered as statistically significant.

## Results

### ALKBH2 is overexpressed in CRC tissues and cells

**Fig. 1:**
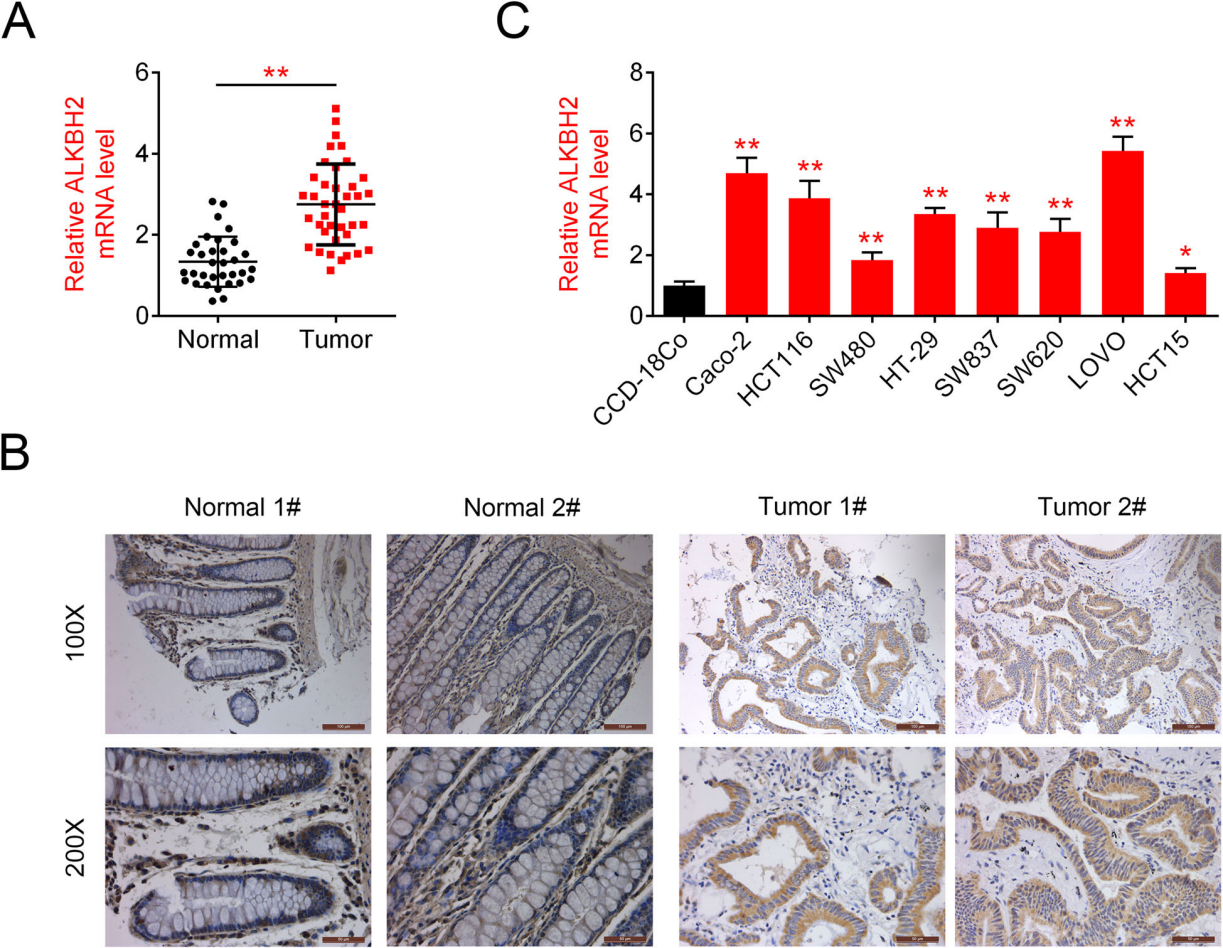
ALKBH2 is upregulated in CRC tissues and cells. **a** Relative ALKBH2 expression in CRC tissues (*N* = 39) and paracarcinoma (*N* = 33), quantified using qRT-PCR assays, **P < 0.01 vs. paracarcinoma. The mRNA levels of ALKBH2 are significantly increased in CRC tissues compared to that of control. **b** Representative IHC images showing ALKBH2 staining in CRC and paracarcinoma, **P < 0.01 vs. paracarcinoma. The percentage of ALKBH2-positive cells is higher in CRC tissues than para-carcinoma. **c** ALKBH2 expression in CRC lines (Caco-2, HCT116, SW480, HT-29, SW837, SW620, LOVO, and HCT15) compared to the colorectal cell (GES-1) as quantified using qRT-PCR assays. ALKBH2 is highly expressed in all colorectal carcinoma cells compared with normal colorectal epithelial cells

The transcript levels and distribution of ALKBH2 in 39 CRC and 33 para-carcinoma tissues were detected by qRT-PCR and immunohistochemistry, respectively. As shown in Fig. 1a, the mRNA levels of ALKBH2 were significantly increased in CRC tissues compared to that of control. Consistently, ALKBH2 staining demonstrated a higher percentage of ALKBH2-positive cells in CRC tissues compared to para-carcinoma control tissues (Fig. 1b). In addition, the expression of ALKBH2 was detected in normal gastric epithelial cell line (CCD-18Co) and different CRC cell lines (Caco-2, HCT116, SW480, HT-29, SW837, SW620, LOVO, and HCT15). qRT-PCR assay showed that ALKBH2 expression was higher in all colorectal carcinoma cells compared with normal colorectal epithelial cells (Fig. 1c). These findings revealed the overexpression of ALKBH2 in CRC tissues and cells.

### ALKBH2 knockdown inhibits CRC cell proliferation

**Fig. 2:**
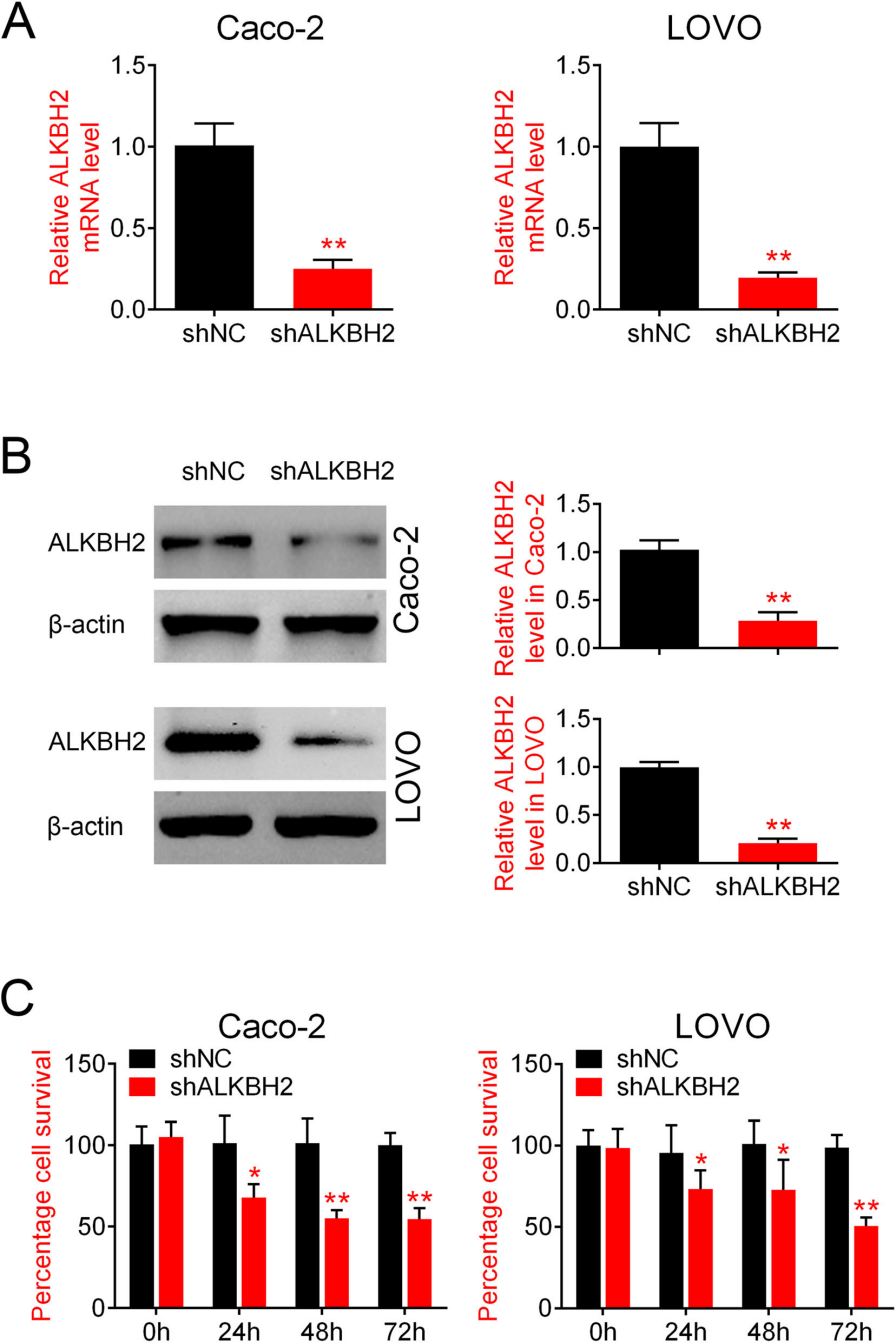
ALKBH2 knockdown inhibits CRC cell proliferation. **a** qRT-PCR was performed to assess the relative ALKBH2 mRNA expression in Caco-2 and LOVO cells transfected with sh-ALKBH2 or sh-NC, **P < 0.01 vs. sh-NC. ALKBH2 mRNA expression is significantly reduced in cells transfected with sh-ALKBH2 compared to sh-NC group. **b** Western blotting was performed to quantify the relative ALKBH2 protein expression in Caco-2 and LOVO cells transfected with sh-ALKBH2 or sh-NC, **P < 0.01 vs. sh-NC. ALKBH2 protein expression is significantly reduced in cells transfected with sh-ALKBH2 compared to sh-NC group C. CCK-8 assay was performed to assess cell viability in Caco-2 and LOVO cells transfected with sh-ALKBH2 or sh-NC, **P < 0.01 vs. sh-NC. Knockdown of ALKBH2 significantly inhibits cell viability in both Caco-2 and LOVO cell lines

Following the finding of ALKBH2 overexpression in CRC tissues and cells, we next sought to determine the effects of ALKBH2 silencing on CRC cell proliferation. ALKBH2 expression was silenced by RNA interference. qRT-PCR analysis and Western blotting results demonstrated that ALKBH2 mRNA and protein expression were significantly reduced in cells transfected with sh-ALKBH2 compared to sh-NC group (Fig. 2a, b). Moreover, CCK-8 analysis showed that knockdown of ALKBH2 significantly inhibited cell viability in both Caco-2 and LOVO cell lines (Fig. 2c). These results suggested that ALKBH2 knockdown inhibited proliferation of CRC cells.

### ALKBH2 knockdown inhibits CRC cell migration and invasion via EMT signaling pathway

**Fig. 3:**
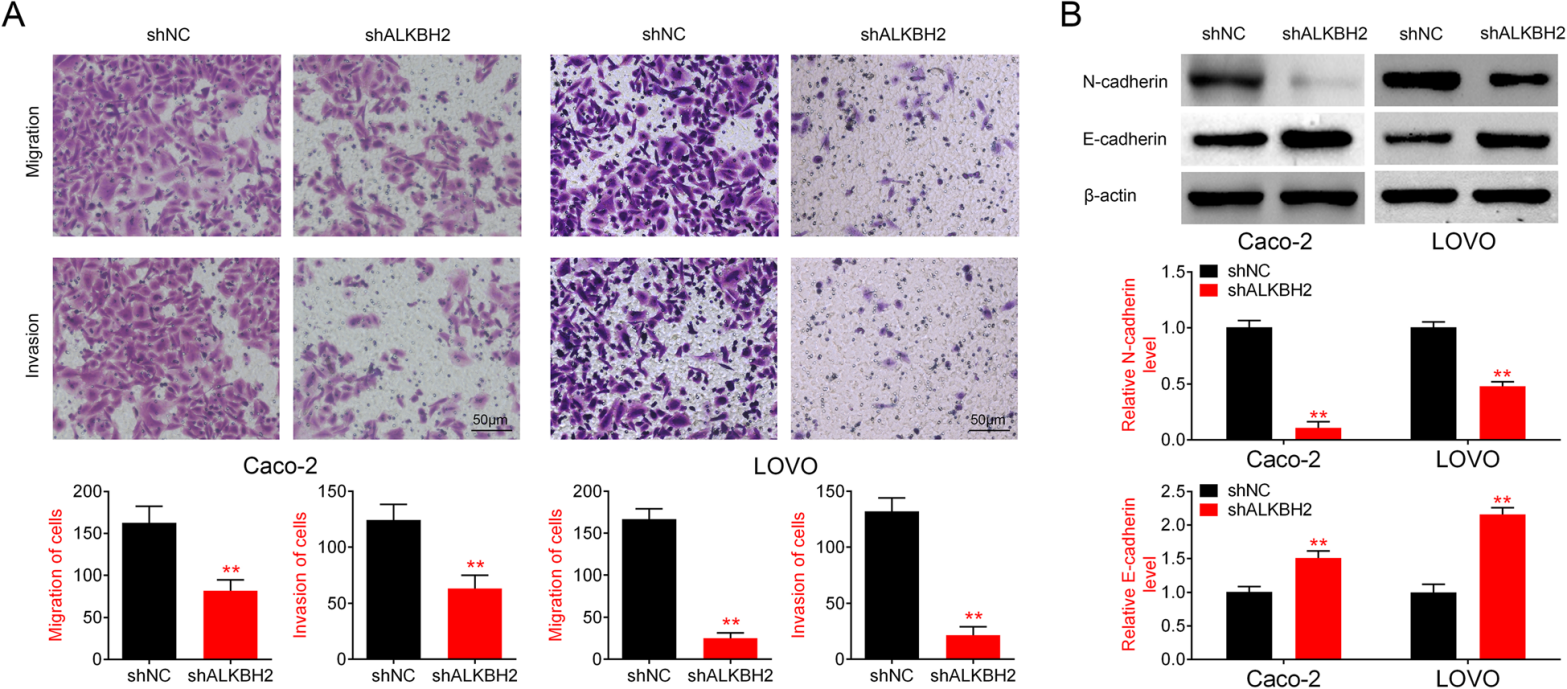
ALKBH2 knockdown inhibits cell migration and invasion of CRC cells via EMT signaling pathway. **a** Cell migration was assessed in Caco-2 and LOVO cells transfected with sh-ALKBH2 or sh-NC, **P < 0.01 vs. sh-NC. The number of migrated and invaded cells is significantly reduced in cells transfected with sh-ALKBH2 compared with the sh-NC group B. Transwell assay was performed to investigate cell invasion in Caco-2 and LOVO cells transfected with sh-ALKBH2 or sh-NC, **P < 0.01 vs. sh-NC. C. Expression changes in EMT-related signaling molecules in cells transfected with sh-ALKBH2 or sh-NC, **P < 0.01 vs. sh-NC. ALKBH2 knockdown increases E-cadherin expression and reduced N-cadherin level

Transwell assays were used to evaluate the effect of ALKBH2 on the migration and invasion of Caco-2 and LOVO cells. Results from the transwell assays revealed a significant reduction in the number of migrated and invaded cells in cells transfected with sh-ALKBH2 compared with the sh-NC group (Fig. 3a). Epithelial-to-mesenchymal transition (EMT) is considered to be an important mechanism for human tumor metastasis [30]. In order to determine the association between ALKBH2 and the EMT pathway, Western blot analysis was performed to measure the expression of EMT-related signaling molecules in Caco-2 and LOVO cells transfected with sh-ALKBH2 or sh-NC. E/N-cadherin switch mediates cancer progression via TGF-β-induced EMT [31]. The results revealed that knockdown of ALKBH2 increased E-cadherin expression and reduced N-cadherin level (Fig. 3b), suggesting that ALKBH2 knockdown suppressed the migration and invasion of CRC cells via EMT signaling pathway.

### ALKBH2 regulates the expression of BMI and the NF- κB pathway

**Fig. 4:**
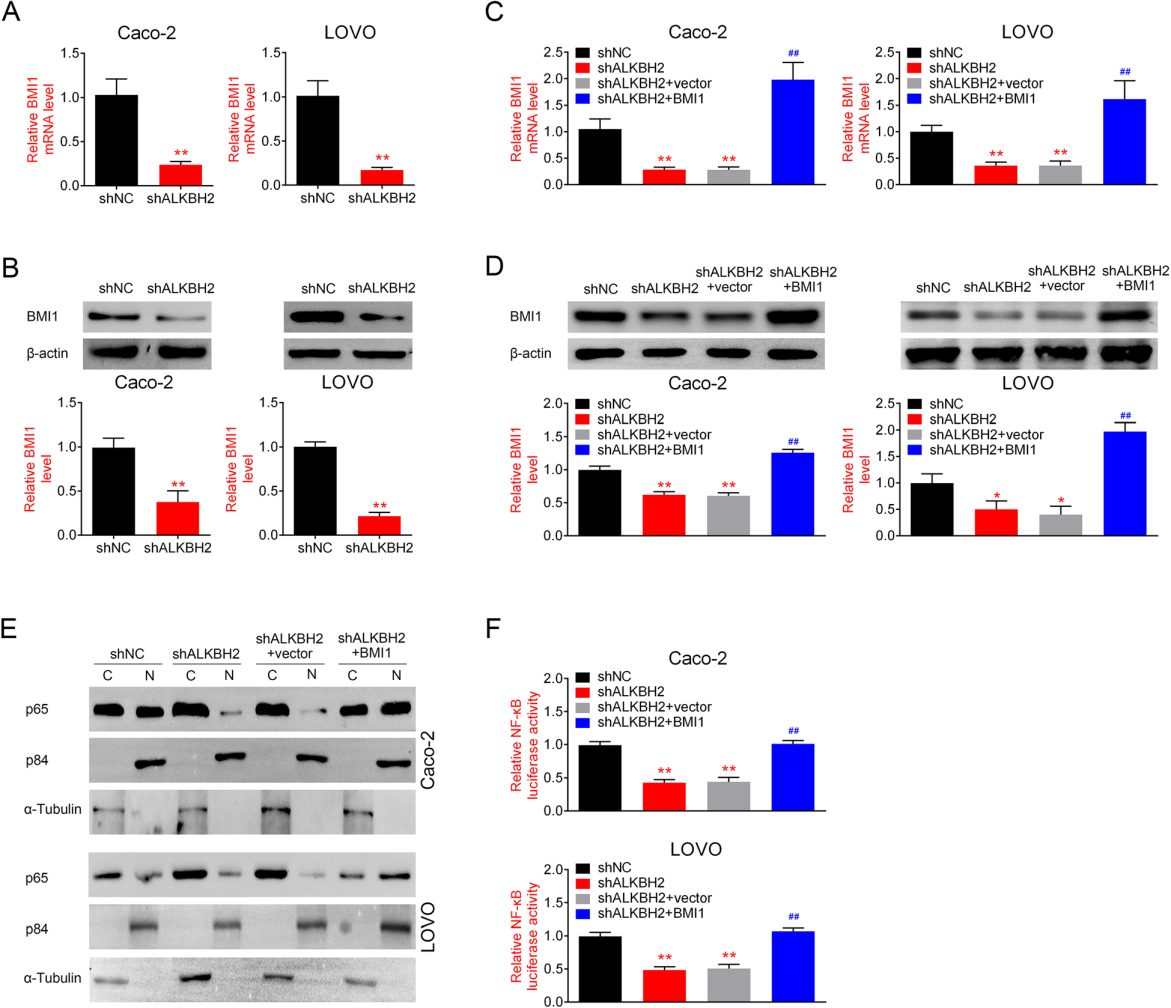
ALKBH2 regulates the expression of BMI1 and the downstream NF-κB pathway. **a** qRT-PCR was performed to determine the relative BMI1 mRNA expression in Caco-2 and LOVO cells transfected with sh-ALKBH2 or sh-NC, **P < 0.01 vs. sh-NC. BMI1 mRNA expression is significantly downregulated after ALKBH2 knockdown. **b** Western blotting was performed to assess the relative BMI1 protein expression in Caco-2 and LOVO cells transfected with sh-ALKBH2, sh-NC, or overexpression of BMI1, **P < 0.01 vs. sh-NC. Compared with the sh-NC group, the expression of BMI1 is downregulated in the sh-ALKBH2 group C. qRT-PCR was performed to measure the relative BMI1 mRNA expression in Caco-2 and LOVO cells after overexpression of BMI1**P < 0.01 vs. shALKBH2+vector. Downregulation of BMI1 is reversed by overexpression of BMI. **d** Western blotting was performed to quantify the relative BMI1 protein expression in Caco-2 and LOVO cells after overexpression of BMI1. **P < 0.01 vs. shALKBH2+vector. Downregulation of BMI1 is reversed by overexpression of BMI. **e** Western blotting was performed to assess the expression of p65 in the cytoplasm and nucleus. Nuclear protein p84 and α-Tubulin were used as nuclear and cytoplasmic marker, respectively, **P < 0.01 vs. sh-NC. C represents the cytoplasm and N represents the nucleus. Knockdown of ALKBH2 by shRNA decreases nuclear accumulation of the NF-κB p65 protein, whereas overexpression of BMI1 increases the content of NF-κB p65 protein in the nuclear component. **f** Immunofluorescent staining of subcellular localization of NF-κB (p65 subunit) in CRC cells, **P < 0.01 vs. sh-NC. The luciferase activities in sh-ALKBH2 treatment group show a significant reduction in nuclear p65, while BMI1overexpression leads to a prominent increase in nuclear p65

To further explore the role of ALKBH2 in the pathogenesis of CRC, we next investigated the effects of ALKBH2 silencing on BMI1 expression and the downstream NF-κB pathway in Caco-2 and LOVO cells. qRT-PCR analysis showed that the mRNA levels of BMI1 were significantly downregulated in ALKBH2-silenced cells (Fig. 4a). Consistently, as shown in Fig. 4b, the protein expression of BMI1 was also downregulated in the sh-ALKBH2 group compared with the sh-NC group. Reduction in BMI1 expression was reversed at both mRNA and protein levels by overexpression of BMI1 (Fig. 4c, d). Since the transcriptional activity of NF-κB is indicated by nuclear accumulation of NF-κB, we next investigated whether ALKBH2 deficiency alters the levels of nuclear NF-κB p65, a catalytic component of NF-κB. As displayed in Fig. 4e, ALKBH2 knockdown by shRNA inhibited nuclear accumulation of the NF-κB p65 protein, whereas overexpression of BMI1 led to opposite effect. The effect of ALKBH2 knockdown on p65 nuclear translocation was further validated using dual-luciferase assays. It was found that the luciferase activity in sh-ALKBH2 treatment group showed a significant reduction in nuclear p65, while BMI1 overexpression led to a prominent increase in nuclear p65 (Fig. 4f). These results suggested that ALKBH2 regulates the expression of BMI1 and the downstream NF-κB pathway.

### Upregulation of BMI1 reverses the effect of ALKBH2 knockdown on CRC cell proliferation and invasion

**Fig. 5:**
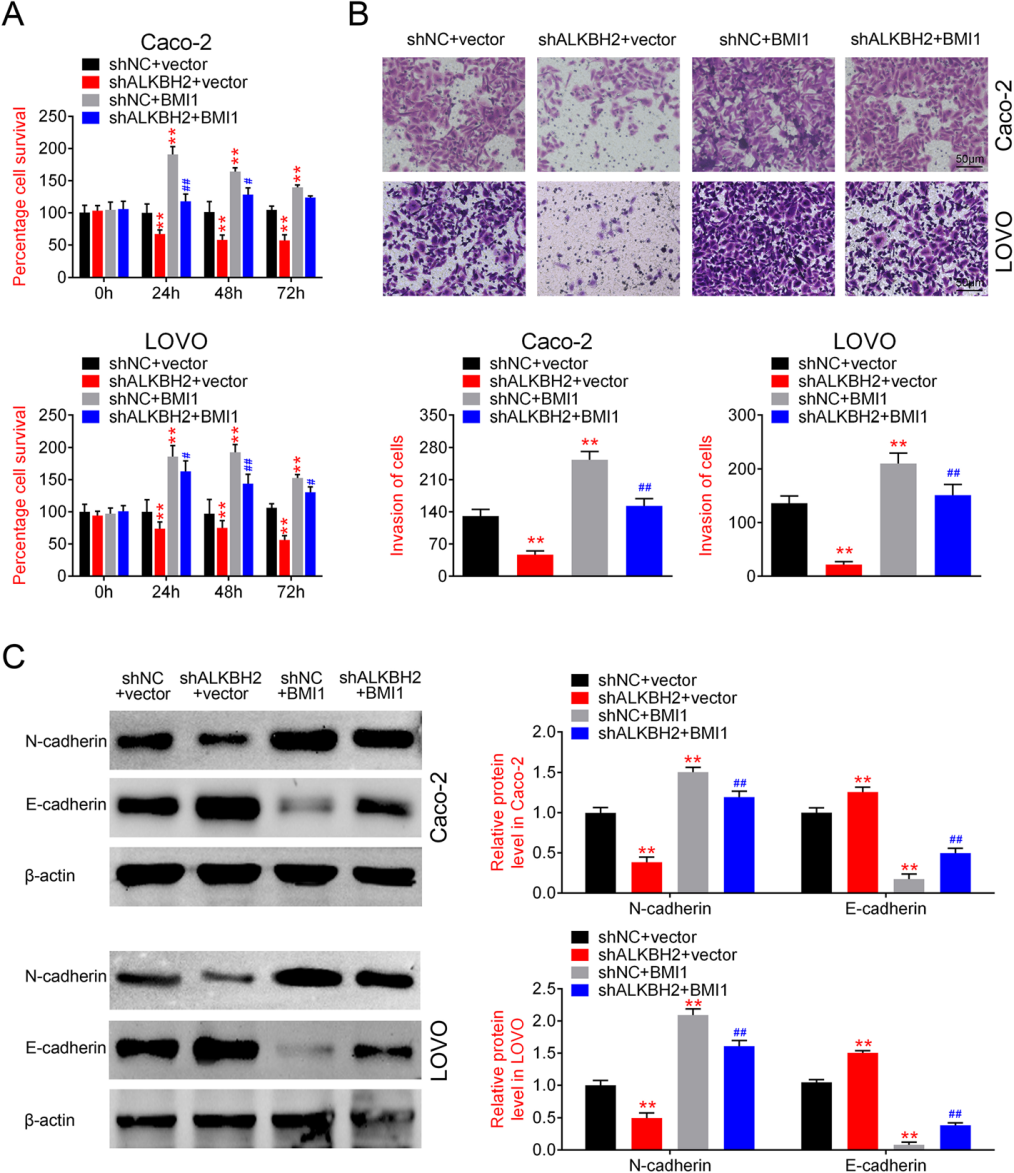
Upregulation of BMI1 reverses the effect of ALKBH2 knockdown on the proliferation and invasion of CRC cells. **a** CCK8 assay was performed to assess cell proliferation in Caco-2 and LOVO cells transfected with sh-ALKBH2, sh-NC, or overexpression of BMI1, **P < 0.01 vs. sh-NC. Histograms show that cell proliferation is decreased in sh-ALKBH2 treatment group, which is reversed by BMI1 overexpression. **b** Transwell assay was performed to detect cell invasion in Caco-2 and LOVO cells transfected with sh-ALKBH2, sh-NC, or overexpression of BMI1, **P < 0.01 vs. sh-NC. Cell invasion decreases in sh-ALKBH2 group, whereas BMI1 overexpression reverses the effect of sh-ALKBH2. C. Expression changes in EMT-related signaling molecules in cells transfected with sh-ALKBH2, sh-NC, or overexpression of BMI1, **P < 0.01 vs. sh-NC. Upregulation of BMI1 increases N-cadherin expression and reduces E-cadherin level

Next, the results from the CCK-8 and transwell assays demonstrated that BMI overexpression reversed the effects of sh-ALKBH2 on cell proliferation (Fig. 5a) and invasion (Fig. 5b) in CRC cells, respectively. In addition, upregulation of BMI1 increased N-cadherin expression and reduced E-cadherin level (Fig. 5c). These results suggested that upregulation of BMI1 could reverse the effects of ALKBH2 silencing on the proliferation and invasion of CRC cells.

## Discussion

CRC is one of the most frequent causes of death worldwide. Thus, identification of new therapeutic target is critical for the therapy of CRC to improve the survival rate in CRC patients. The *ALKBH2* gene is involved in DNA repair and is expressed in many malignancies. However, the role of *ALKBH2* in the pathogenesis of CRC remains unclear. This study explored for the first time the functions and clinical significance of ALKBH2 in CRC.

The oxidases of the AlkB family represent a new class of DNA base repair enzymes that employ oxidative dealkylation mechanism. The ALKBH protein families are highly expressed in all kinds of human malignant tumors, and they are implicated in tumor progression and development. Shimada et al. reported that ALKBH8 promotes the progression of human urinary tract epithelial cancer by downregulating NAD(P)H oxidase-1(NOX-1) and by subsequently activating c-jun NH(2)-terminal kinase (JNK) and p38 NADPH oxidase 1-dependent reactive oxygen species to induce apoptosis [32]. Moreover, upregulation of ALKBH3 is implicated in the development of non-small cell lung cancer and prostate cancer [33]. However, ALKBH2 has been less studied in tumors and has not been studied in CRC. This is the first study that investigated the role of ALKBH2 in CRC. The findings from this study have demonstrated that ALKBH2 was overexpressed in CRC, and it promoted cell proliferation, metastasis, and invasion. In addition, we have shown that ALKBH2 silencing inhibited EMT, which increased E-cadherin expression and reduced N-cadherin level. Increasing data have indicated that cells with EMT phenotype consist of a plentiful of tumor stem cells with cytotoxic injury-induced repair signals, proposing a biological connection between EMT and DNA repair system. EMT offers the most suitable microenvironment for DNA repair molecules to carry out their functions. Interestingly, the findings from this study have also shown that ALKBH2 knockdown decreased BMI1 expression and inhibited nuclear accumulation of the NF-κB p65 protein, as well as the luciferase activity of NF-κB p65.

The carcinogenic features of BMI1 have linked BMI1 expression to cancer development and clinical prognosis of human tumors (Table 2). Studies have demonstrated that BMI1 can regulate cancer initiation and cell transformation [16, 34]. Li et al. have reported that BMI1 exerts glioma cells apoptotic resistance via the activation of NF-κB-mediated pathway [35]. Our study showed a concurrent decrease in the expression of BMI1 and nuclear NF-κB (the p65 submit), which demonstrated that BMI1 reinforced NF-κB transcriptional activity through accelerating nuclear translocation of NF-κB. In addition, upregulation of BMI1 reversed the impact of ALKBH2 knockdown on cell proliferation, invasion, and EMT in CRC cells. This has not only confirmed our results, but also supports the mechanistic data, indicating that ALKBH2 downregulation inhibited BMI1 expression and suppressed CRC proliferation. Increased ALKBH2/BMI1 expression is closely associated with transformation from a noninvasive to an invasive phenotype. Molecular therapy focusing on ALKBH2 may represent an attractive tool to achieve successful therapy in the treatment of human CRC.

**Table 2:** Clinical features of 33 tumor samples

Characteristics	Total of patients	ALKBH2 Low expression (≤ median)	ALKBH2 High expression (> median)	*P* value
Number	33	17	16	
Gender				0.392
Male	21	12	9	
Female	12	5	7	
Ages (years)				0.325
< 65	22	10	12	
≥ 65	11	7	4	
Tumor site				0.114
Colon	21	13	8	
Rectum	12	4	8	
Tumor size(cm)				0.387
< 5	17	10	7	
≥ 5	16	7	9	
Pathological T category				0.009*
T1-T2	20	13	5	
T3-T4	13	4	11	
Lymph node metastasis				0.026*
N0	26	16	10	
N1-N2	7	1	6	
Distant metastasis				0.028*
M0	30	17	12	
M1	3	0	4	
TNM stage				0.021*
I–II	21	14	7	
III–IV	12	3	9	
Differentiation				0.353
Well/moderate	20	9	11	
Poor	13	8	5	

*p < 0.05

## Conclusion

In conclusion, this study showed that suppression of ALKBH2 alleviated malignancy in CRC by regulating BMI1-mediated activation of NF-κB pathway. ALKBH2 may serve as a potential target for the development and progression of human CRC.

## Data Availability

All data generated or analyzed during this study are included in this published article.

## Abbreviations

CRC: Colorectal cancer
EMT: Epithelial-mesenchymal transition
BMI1: B cell-specific Moloney murine leukemia virus integration site 1
dsDNA: Double-stranded DNA
PcG: Polycomb-group
DMEM: Dulbecco’s modified Eagle medium
FBS: Fetal bovine serum
CCK 8: Cell Counting Kit 8
qRT-PCR: Quantitative real-time PCR
HCC: Hepatocellular carcinoma
